# Deep learning enhanced prediction framework for bio oil yield from organic solid waste with chemically informed features

**DOI:** 10.1038/s41598-026-43604-7

**Published:** 2026-03-16

**Authors:** Shahad Almansour, Lulwah M. Alkwai, Kusum Yadav, Debashis Dutta

**Affiliations:** 1 Applied College, University of Ha’il, Ha’il, Kingdom of Saudi Arabia; 2 College of Computer Science and Engineering, University of Ha’il, Ha’il, Kingdom of Saudi Arabia; 3 AI for All Initiative, Riyadh, Kingdom of Saudi Arabia

**Keywords:** Bio-oil yield, Deep learning, Pyrolysis, Biomass valorization, Feature engineering, Thermochemical modeling, Chemistry, Energy science and technology, Engineering, Environmental sciences, Materials science

## Abstract

Accurate prediction of bio-oil yield from pyrolysis of organic solid waste is still the main problem in the field of biomass valorization, mostly because of high variability in the composition and the heterogeneity of experimental conditions reported in the literature. Machine-learning techniques described in existing literature frequently depend on datasets that are too narrow, have limited feature representations, or shallow models that cannot sufficiently capture nonlinear thermochemical interactions that control devolatilization and liquid formation. In this study, the present study develops a deep learning-based predictive framework that addresses these gaps by using a harmonized dataset of 245 samples derived from diverse biomass sources and pyrolysis conditions. The model employs a chemically guided feature-engineering strategy that uses elemental ratios, ash-corrected volatility, and an energy-density index, followed by Variance Inflation Factor (VIF)-driven feature selection to lessen multicollinearity while keeping the mechanistic relevance. The resulting Hybrid DNPO achieves an R^2^ of 0.980 and an RMSE of 1.14 on new data, making it better than all benchmark regression models, including Light Gradient Boosting (LGB). The results show that a thermochemically grounded, chemically informed deep neural prediction framework is proposed, integrating feedstock compositional descriptors and operating conditions to predict bio-oil yield from organic solid waste pyrolysis, significantly improving the performance of prior models from the literature, and can serve as a reliable tool for guiding experimental design, biomass screening, and process optimization in eco-friendly bio-oil production. By leveraging a larger, diverse dataset and advanced feature engineering, the methodology also offers a solid foundation for future studies aiming to enhance the accuracy and robustness of biomass-to-biofuel conversion predictions.

**Supplementary Information:**

The online version contains supplementary material available at 10.1038/s41598-026-43604-7.

## Introduction

The use of energy derived from fossil fuels is increasing as a result of several developing economies and a growing population. As a result, many nations’ fossil fuel supplies are being depleted, greenhouse gas emissions are rising, and fuel prices are rising^1,2^. Potential solutions to address the aforementioned issues and lessen reliance on fossil fuels geographically might include renewable energy sources^3^. Potential substitutes include a range of renewable energy sources, such as geothermal, hydro, biomass, wind, and solar energy. Of these, the most sustainable and promising energy source that can be used to produce chemicals and energy instead of fossil fuels is biomass energy, also known as bioenergy^4,5^. A renewable organic substance produced mainly by vegetation, biomass comprises sewage sludge, food waste, municipal solid waste, and agricultural and forest residue^6,7^. It can be subjected to biochemical and thermochemical conversion processes to produce solid, liquid, and gaseous materials. There have been tremendous technological advancements by scientists in the past several decades, creating inexpensive thermochemical processes with relatively efficient conversion and little pretreatment^8,9^.

One of the key points of the recent research is experimental biofuel production and the integration of machine learning (ML) to improve yield prediction, process optimization, and reaction condition interpretation^10–12^. Ramalingam et al.^13^ experimented on biodiesel synthesis from waste cooking oil with a CaO catalyst made from eggshells and combined 16 transesterification experiments with boosted ML models. Their analysis through ML models identified catalyst concentration and methanol-to-oil molar ratio as the two variables that most strongly predicted biodiesel yield. CatBoost was the algorithm that achieved the highest predictive accuracy among several tested, thus it enabled reliable data-driven optimization. Reduction of CO and smoke emissions for CaO-based biodiesel was confirmed by engine tests, apart from the laboratory conditions; thus, both environmental benefits and practical applicability were demonstrated. In addition to Lee et al.^14^’s extensive review of ML techniques in the context of bio-oil production from the pyrolysis of lignocellulosic biomass, they examined the applicability of these methods in related experimental computational integrations. Their evaluation revealed that regression-based models, especially random forest, artificial neural networks, gradient boosting, support vector regression, and linear regression, are the leading methods for prediction in this field. They pointed out that the output of the models often corresponds well to the experimental results; however, due to limited datasets and the necessity for better feature extraction, there are still some challenges. They stated that their research has identified that current ML models still have the potential to be further developed for more precise forecasting of pyrolysis performance and bio-oil properties.

Liu et al.^15^ moved the frontier forward by the application of ML to Hydrothermal co-liquefaction of sewage sludge and algal biomass. They utilized Gradient Boosting Regression and Random Forest to model yield, nitrogen content, and energy recovery rate. Their multitask learning approach achieved an impressive average test R^2^ of 0.84, pointing to temperature as the most critical variable; the optimal value was around 350 °C for both maximum yield and energy recovery, and about 280 °C for minimum nitrogen content. The experiment validation also shows low prediction errors; thus, the capability of ML to guide co-liquefaction process optimization is convincingly demonstrated, an area that has been mostly neglected so far. In another contribution, Lee et al.^16^ examined ML advancements in pyrolysis-based bio-oil prediction, again finding that random forest, neural networks, gradient boosting, SVR, and linear regression remain the dominant approaches. They highlighted consistent agreement between ML predictions and experiments, while reinforcing concerns surrounding limited datasets and insufficient algorithmic diversity. Iweka et al.^17^ employed Box–Behnken Design alongside ML to optimize bio-oil production from ripe pawpaw seeds. Their ML model outperformed the statistical design in predictive accuracy, achieving a maximum yield of 23.97 wt% and producing bio-oil with physicochemical properties suitable for biodiesel applications. Djandja et al.^18^ focused on predicting bio-oil yield from lignocellulosic biowaste using eXtreme Gradient Boosting, incorporating biochemical components and operating factors. They emphasized biomass conversion as a key variable, achieving high prediction accuracy through a developed model and graphical user interface. Onokwai et al.^19^ focused on predicting bio-oil yield from Cocos nucifera pyrolysis using hybrid models (PSO-ANFIS and GA-ANFIS) and traditional RSM, emphasizing the optimization of parameters like temperature and heating rate, but did not specifically address organic solid waste. Katongtung et al.^20^ focused on predicting biocrude oil yields from biomass hydrothermal liquefaction using a gradient tree boosting machine model and principal component analysis, but it does not specifically address a prediction framework for bio-oil yield from organic solid waste.

While machine learning has been progressively utilized in biomass conversion research, a few fundamental limitations still exist. Normally, studies rely largely on small, experiment-specific datasets, which in turn limit the generalizability of traditional models and their capacity to represent various biomass types or reactor conditions^21,22^. In fact, most ML-based research has narrowly focused on feature sets, overlooking key physicochemical interactions governing pyrolysis. Overviews have consistently noted insufficient feature engineering, limited cross-study variability, and a lack of advanced deep learning approaches, all of which reduce predictive robustness for complex thermochemical pathways^23^. Besides that, multicollinearity and experimental heterogeneity persist. Since datasets based on literature sources mix different feedstocks, reactors, and analytical methods, models developed without systematic feature reduction or variance assessment often become unstable. There are very few papers that incorporate engineered physicochemical indices reflecting ultimate reactivity, leading to models that ignore the deeper chemical drivers of bio-oil formation, such as hydrogen availability, energy density, and ash-related volatilization effects^24^.

The gaps in the current study are addressed by the present research, which develops a hybrid deep learning–optimization framework based on a comprehensive, literature-derived pyrolysis dataset. The novelty of this work is due to three major contributions:

Deep Neural Network Architecture for Bio-Oil Yield Prediction: The current work moves beyond linear regression or classical tree-based methods used in previous research by implementing a deep neural network that is properly regularized and capable of representing multilevel nonlinear relationships between elemental composition, proximate properties, and operating conditions. As a result, predictive fidelity is greatly improved across biomass classes of different natures. Chemically Informed Feature Engineering and VIF-Driven Selection: A structured feature engineering strategy was implemented that included thermochemically meaningful variables such as H/C, ash-corrected volatile content (VC*), and an energy-density index (EDI). Along with detailed variance inflation analysis, this method yields a compact 9-feature input matrix that not only balances statistical independence but also provides mechanistic interpretability - an improvement that was largely ignored in previous ML biofuel studies.

## Dataset construction and harmonization

### Dataset establishment and preprocessing

The data set for this research was compiled from a series of experiments on bio-oil production via biomass pyrolysis^25^, which previously published experimentally derived organic solid waste upgrading data under consistent techno-economic and life-cycle assessment frameworks. The primary collection comprises 245 samples, each representing a different set of biomass properties, pyrolysis process conditions, and bio-oil yield. Besides, the parts of the detailed numerical values for all the samples were taken straight from the support information of the original study, so that they can be consistent with the results that have been reported experimentally. In addition, every numerical record includes a full set of variables that are necessary for machine learning models: ultimate analysis (C, H, N, O), proximate analysis (ash, fixed carbon, volatile content), and pyrolysis temperature, together with the bio-oil yield on a dry basis of the organic phase that has been reported. Furthermore, the initial data set covers a wide range of biomass types, including herbaceous materials, lignocellulosic biomass, agricultural residues, and algae, to reflect the variation observed in real thermochemical conversion systems. The data extracted from the scientific articles have two categories of unavoidable uncertainty inherently: (1) Measurement uncertainty, in some studies’ certain proximate parameters (e.g., fixed carbon) are reported by difference rather than direct measurement; and (2) Experimental uncertainty, which is caused by variations in reactor configurations, heating methods, and condensers, or feedstock handling. They account for the difficulties that are typical in research on biomass pyrolysis and should be considered in the development of the model.

To ensure data quality and consistency, the following inclusion criteria were applied: (i) availability of complete proximate and ultimate analyses; (ii) clearly reported pyrolysis operating conditions, including temperature, heating rate, and residence time; and (iii) explicit reporting of bio-oil yield. Samples with missing compositional variables, undefined operating parameters, or ambiguous yield measurements were excluded. Fixed carbon values reported by difference were retained as provided in the source study.

The finalized dataset, including all input variables and target outputs used in the present modeling framework, is provided as a Supplementary File in Excel format to promote transparency, reproducibility, and reuse by the research community.

**Fig. 1:**
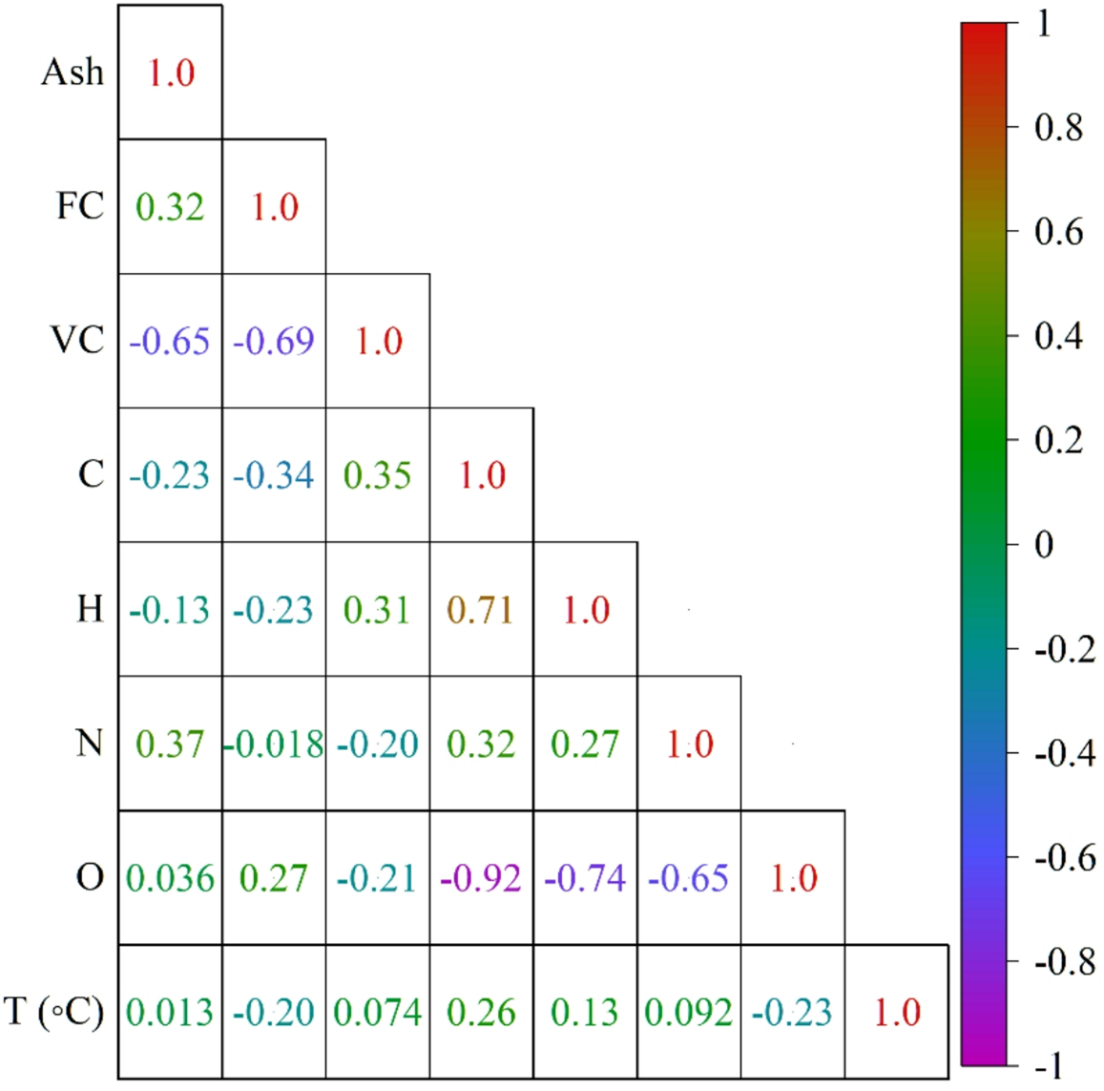
PCC heatmap displaying linear correlations among all variables (C, H, N, O, ash, FC, VC, temperature, REF index, and bio-oil yield).

**Fig. 2:**
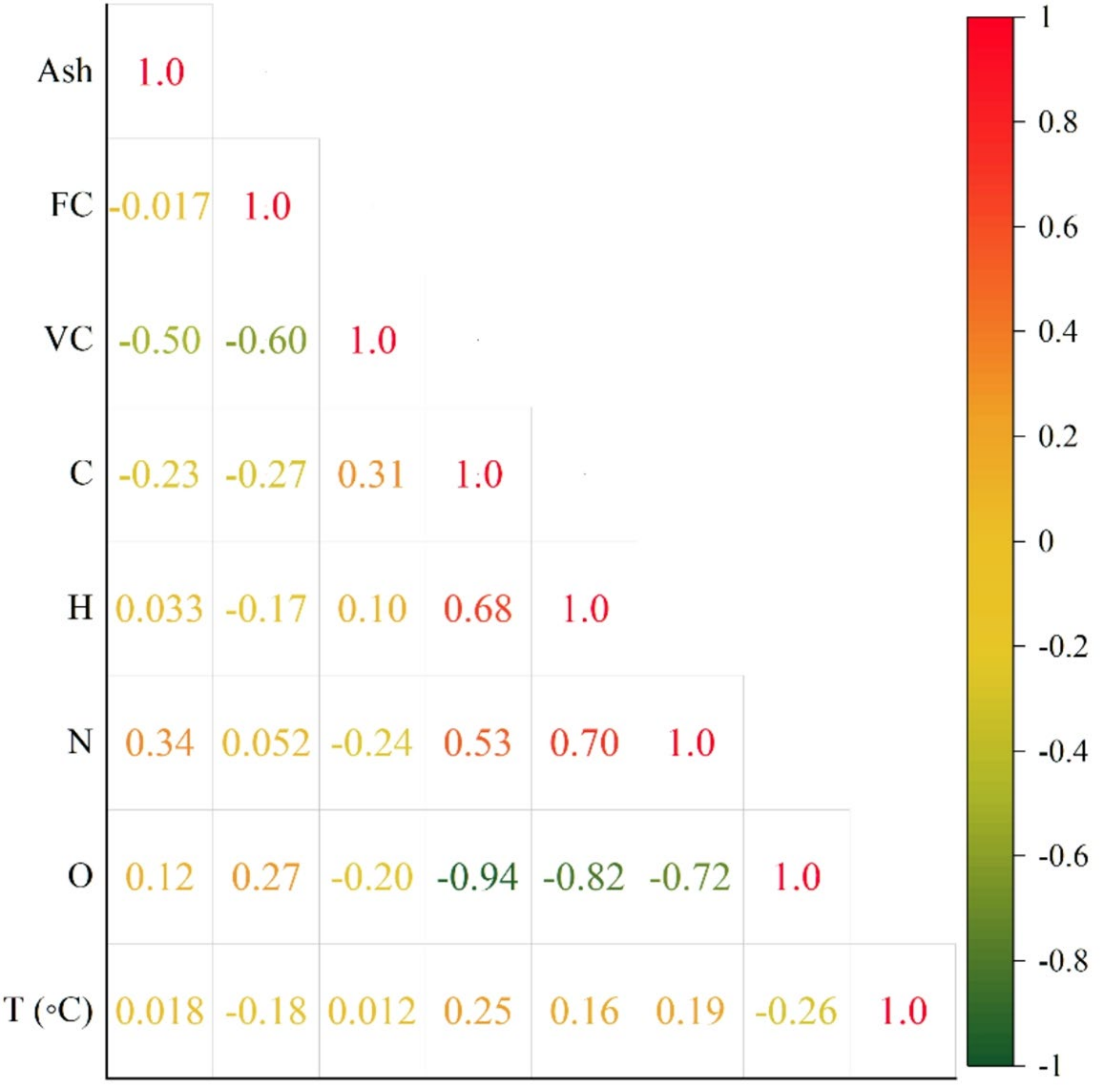
Correlation coefficient analyses based on Spearman.

First of all, the dataset was scrutinized for missing data, outliers, unit inconsistencies, and strange measurements to guarantee that machine-learning models receive well-organized input. In order to keep the full chemical and operational variety of the original literature, no samples were discarded. All bio-oil yields were converted to a dry, organic-phase basis in order to remove any differences that varying moisture or aqueous-phase fractions might have caused. To begin with, the Pearson correlation coefficient (PCC) was used to evaluate the strength and direction of linear relationships between the features in the dataset (Fig. 1). This was followed by the creation of a correlation matrix to identify potential multicollinearity, which is particularly important for models sensitive to linear dependencies. While the PCC provides valuable insights into linear relationships, nonlinear models such as Light Gradient Boosting Machines (LGBM) and Deep Neural Networks (DNN) benefit from recognizing nonlinear feature interactions. Thus, additional metrics were employed to capture nonlinear dependencies. Spearman’s rank correlation coefficient was calculated to measure the strength of monotonic relationships, providing a broader assessment of nonlinear trends, as shown in Fig. 2. This combination of linear and nonlinear metrics offers a comprehensive view of the relationships between the features in the dataset, ensuring that both types of dependencies are accounted for.

### Feature engineering and data transformation

Considering the extreme variability of biomass and pyrolysis systems, obtaining features plays a major part in elevating the model’s strength and taking out patterns that make sense physically. To facilitate prediction, several transformations were introduced while retaining the chemical significance of the original dataset.

#### Feature scaling and normalization

Every numerical variable was Min-Max scaled using the Min-Max scaling method to the range [0, 1]. This is necessary for optimization algorithms like NMS and PO to work on a uniform search space and also to prevent any single variable from dominating the loss gradients. In models that are intrinsically capable of handling unscaled features (e.g., LGBM), both the raw and scaled versions were tested, and scaling led to more stable optimization convergence for hybrid models.

#### Derived chemical indices

In an effort to make the model more chemically interpretable, three derived physicochemical features were introduced, with the features being based on the well-known relationships in pyrolysis chemistry: (1) H/C Ratio: One of the indicators of hydrogen richness. Generally, higher H/C ratios are associated with more reactive volatiles and better bio-oil quality. (2) O/C Ratio: A parameter to measure oxygen contribution. A high O/C is usually associated with increased production of oxygenated compounds and lower heating value. (3) Volatile-to-Fixed Carbon Ratio (VC/FC): One of the most common pyrolysis reactivity indices. Generally, higher ratios signal more devolatilization and thus higher liquid yields. (4) Ash-Corrected Volatile Content (VC*): It is a portion that accounts for mineral content that impedes devolatilization. (5) Energy Density Index (EDI): A rough estimate of energy content per unit mass (very similar to ultimate-analysis-based HHV correlations).

Table 1 details all the input variables for the study. It includes not only the original experimental measurements but also the features derived during preprocessing. The original variables proximate analysis, ultimate analysis, and pyrolysis conditions portray the basic physicochemical descriptors that have been used traditionally to explain the variation in bio-oil production. Nevertheless, as these raw features often have nonlinear and interacting effects, the study introduced additional engineered variables to improve the representational power of the machine-learning models. The engineered features were picked based on solid thermochemical principles. Ratios like H/C and O/C provide an account of hydrogen richness and oxygenation level, respectively, which are closely related to devolatilization behavior and the stability of pyrolysis vapors. The VC/FC ratio thus serves as a reactivity index that shows the equilibrium between volatile release and char formation, whereas the ash-corrected volatile content (VC*) is a performance measure that accounts for both catalytic and inhibitory effects of mineral matter. Further, the incorporation of an energy-density-based index (EDI) essentially folds the elemental composition into a single metric that mirrors the energetics of thermal decomposition, which are anticipated.

**Table 1:** Original and engineered input features: units, physical meaning, expected influence on bio-oil yield, and derivation formula.

Variable name	Units	Physical meaning	Expected influence on bio-oil yield	Formula/definition
Original				
Ash	wt%	Inorganic mineral residue remaining after combustion; inhibits devolatilization and catalyzes secondary cracking	Generally, a negative influence; higher ash lowers bio-oil yield	–
Fixed Carbon (FC)^26^	wt%	Solid carbonaceous residue after devolatilization; an indicator of char formation tendency	Typically negative; higher FC leads to more char formation instead of liquids	–
Volatile Content (VC)^26^	wt%	Fraction that vaporizes during pyrolysis; directly linked to liquid formation	Strong positive influence; high VC improves bio-oil yield	–
Carbon (C)	wt%	Basic structural element of organic matter; contributes to energy density	Weak direct influence; impact captured better through derived ratios	–
Hydrogen (H)	wt%	Contributes to hydrocarbon formation and liquid stability	Mild positive effect; promotes volatiles and stabilizes liquids	–
Nitrogen (N)	wt%	Source of nitrogenous compounds; affects oil quality more than yield	Generally neutral to yield; may introduce noise	–
Oxygen (O)	wt%	Source of oxygenates in vapors; correlates with biomass reactivity	Mixed effect; high O increases volatiles but lowers oil stability	–
Pyrolysis Temperature (T)	°C	Thermal severity controls devolatilization and secondary cracking	Non-linear influence; moderate temperatures maximize yield	–
Engineered				
H/C Ratio^27^	mol/mol	Degree of hydrogen richness indicates the volatile evolution tendency	Positive; higher H/C generally supports higher liquid yield	$H/C=H/C$
O/C Ratio^27^	mol/mol	Relative oxygen content: an indicator of oxygenated compounds	Often negative; higher O/C may promote char and reduce stable liquids	$O/C=O/C$
VC/FC Ratio^28^	–	Key reactivity index: high values indicate higher devolatilization potential	Strong positive correlation with liquid formation	$VC/FC=VC/FC$
Ash-Corrected VC (VC*)^29^	wt%	Adjusts volatile content for the inhibitory effect of ash	Positive: better representation of the actual volatile fraction	$V{C}^{*}=VC\times(1-Ash/100)$
Energy Density Index (EDI)^30^	Arbitrary units	Estimated energy content based on ultimate analysis; linked to condensable products	Positive; higher EDI often correlates with higher-quality pyrolysis vapors	$EDI=(32.8\times C)+(142\times H)-(15.9\times O)$

#### Sensitivity analysis (FAST method)

To assess the influence of individual and interacting input variables on the model’s output, we employed the Fourier Amplitude Sensitivity Test (FAST). FAST is a global sensitivity analysis technique that quantifies the contribution of each input variable to the total variance of the output, providing insights into how each feature affects bio-oil yield prediction within our deep learning model. FAST decomposes the input space into Fourier series components, enabling quantification of each variable’s influence via a set of amplitude coefficients. The analysis was performed by:

1. **Sampling input variables**: The input variables, which include both original and engineered features, were sampled over a predefined range based on the training data distribution.
2. **Computing sensitivity indices**: The total variance in the output was decomposed into contributions from each input feature, as well as interactions between features. This was done by calculating the first-order and total sensitivity indices for each feature.
3. **Model evaluation**: The model was evaluated using the same 9-feature matrix as described in section 2.2.4. The sensitivity indices were derived by applying the FAST algorithm over the entire model output range.

The FAST sensitivity indices provided valuable insights into the relative importance of each input feature and their interactions with others. High first-order sensitivity indices indicate that the corresponding variable has a strong, direct influence on the bio-oil yield prediction, whereas significant total sensitivity indices suggest that interactions between variables may also play a crucial role in model behavior. These results help identify the key thermochemical and process variables that most impact bio-oil yield and provide a better understanding of the underlying physical processes captured by the model.

### Uncertainty identification and mitigation

The compiled dataset integrates results from multiple independent studies, inherently introducing two categories of uncertainty: measurement-related and experimental. Measurement uncertainty arises primarily from proximate analysis practices, where fixed carbon is commonly calculated by difference rather than directly measured, leading to cumulative propagation of analytical errors. Experimental uncertainty originates from variations in reactor configuration, heating methodology, condensation systems, and feedstock preprocessing, all of which influence vapor residence time, secondary reactions, and liquid recovery efficiency.

These uncertainties were identified through systematic inspection of reporting methodologies across the source literature, including reactor types (fixed-bed, batch), heating modes, and analytical procedures.

Rather than explicitly modeling uncertainty distributions—limited by inconsistent error reporting in the literature—the present study mitigates these effects through: (i) harmonization of operating parameters into unified descriptors; (ii) chemically informed feature engineering to encode intrinsic biomass reactivity independent of reactor-specific artifacts; (iii) variance inflation factor–based feature selection to suppress correlated noise; and (iv) cross-validated deep learning with regularization to promote generalization across heterogeneous experimental conditions. This strategy enables the model to learn invariant thermochemical relationships while implicitly absorbing inter-study variability.

## Predictive modeling framework

### Overview of the modeling strategy

The predictive modeling framework in this paper aimed to predict bio-oil yield from a condensed set of engineered physicochemical features that characterize both the compositional and thermochemical nature of biomass. As bio-oil production is a result of nonlinear interactions, e.g., temperature-dependent devolatilization, ash-catalyzed secondary cracking, and elemental reactivity, two complementary predictive models were implemented: a Deep Neural Network (DNN) due to its universal function-approximation capability, and Light Gradient Boosting (LightGBM) as a leading tree-based model for structured tabular data. To exhaustively search for model hyperparameters, two global optimization heuristics, the Non-Monopolized Search (NMS) and Puma Optimizer (PO), were used separately for both algorithms. The models’ performances were measured by RMSE, MAE, R^2^ and etc. on stratified train–validation–test splits to guarantee an unbiased assessment of the different experimental sources.

### Deep Neural Networks (DNN)

#### Architecture

The deep neural network was formulated to represent complex nonlinear interactions between biomass composition, thermochemical properties, and pyrolysis operating conditions. Ultimately, three hidden layers formed the architecture, each aimed at learning progressively higher-level feature interactions. The first hidden layer had 64 neurons, the second 32, and the third 16. This tapered structure invites the network to model broad compositional trends before learning localized nonlinearities, thus facilitating coarse-to-fine abstraction. The ReLU (Rectified Linear Unit) activation function was used in all hidden layers, chosen for its ability to handle nonlinear thermochemical responses while avoiding the vanishing gradient problem typical of sigmoidal activations. To enhance the stability and convergence of samples derived from the literature and heterogeneous data, initialization was applied to all weights. Dropout layers (with a dropout rate of 0.2) were placed after the first and second hidden layers to counter overfitting by preventing co-adaptation, whereas batch normalization was applied before each activation to alleviate internal covariance shift and facilitate faster learning. A single neuron with linear activation constituted the output layer to estimate continuous bio-oil yield^31,32^.

#### Scientific rationale

Essentially, the deep neural network (DNN) was adopted to model the inherently nonlinear pyrolysis chemistry. In this case, the bio-oil yield is the result of the interaction of the carbon–hydrogen–oxygen composition, ash-induced catalytic effects, and temperature-dependent volatilization kinetics. Typically, regression models fail to capture these multi-axis interactions, but a DNN can easily represent high-dimensional, non-additive patterns. In fact, the network’s depth enables it to discover subtle interactions between volatile/fixed carbon ratios and operating temperature, while engineered features, such as H/C or energy-density indices, enable the model to deduce the underlying thermochemical pathways. Moreover, the DNN can leverage its generalization across heterogeneous data sources because the dataset consolidates results from numerous studies, and REF-stratified partitions guide training. Hence, the DNN is a more accurate and interpretable model that offers a physically consistent, more flexible mapping from biomass properties to pyrolysis yield than simpler models.

### Light Gradient Boosting (LGB)

#### Model description

Light Gradient Boosting Machine (LightGBM) is a set of gradient boosting algorithms aimed at quick and accurate prediction of structured tabular data. It achieves accuracy improvement by adding decision trees one after another, where each tree is fit to the residuals of the previous ensemble, following the principle of gradient boosting. LightGBM, however, features a leaf-wise growth strategy instead of the traditional level-wise tree growth; thus, it grows the leaf that yields the highest loss reduction each time. Therefore, this process allows the trees to be deeper and hence capable of capturing drastically nonlinear boundaries characteristic of biomass pyrolysis behavior. To speed up training and lower memory usage, LightGBM adopts histogram-based binning in which the continuous features are first transformed into a fixed number of bins. This saves the computation time while maintaining the sufficient detail required for the modeling of complex thermochemical relationships. LightGBM achieves this by the combination of gradient boosting, leaf-wise tree construction, and efficient histogram binning, and hence it is the one to be considered for datasets with mixed-scale engineered physicochemical features and nonlinear interactions^33,34^.

#### Scientific justification

Reasonably, LightGBM can act as a powerful supplementary method to deep neural networks due to its capability to deal with mixed-scale tabular data, that is, a combination of elemental compositions, proximate analysis variables, engineered ratios, and operating conditions. The leaf-wise boosting mechanism of this tool is an excellent way of naturally absorbing thermochemical patterns like the effect of volatile content, ash behavior, or H/C ratio on bio-oil yield without almost any data being required for training. Thus, LightGBM is exceptionally useful for literature-derived datasets of a moderate size. Besides, LightGBM allows straightforward interpretation from feature importance measures that provide knowledge of the physicochemical variables that most strongly influence pyrolysis. This interpretive capability fosters scientific understanding of biomass–temperature–composition interactions and offers a transparent standard against which the more flexible but less inherently interpretable DNN can be compared.

### Optimization methods

Hyperparameter optimization is a major factor enabling the success of machine learning models, especially when applied to non-linear thermochemical systems having various compositional ranges and originating from heterogeneous experiments. Two global-search heuristics, namely the Non-Monopolized Search (NMS) algorithm and the Puma Optimizer (PO), were used in this work to adjust the main hyperparameters of both the DNN and LightGBM models. The selection of these optimizers was not for the sake of making a contribution, but rather as a means of systematic searching to facilitate balanced exploration and exploitation of the high-dimensional parameter space governed by the engineered physicochemical features.

#### Non-Monopolized Search (NMS)

NMS is a global optimization strategy based on a population that aims to avoid the dominance of any single solution at the early stage of the search. It keeps diversity among the candidate solutions and lessens the chance of premature convergence; thus, it is a good choice for hyperparameter landscapes with multiple local minima. The mentioned feature of NMS is very instrumental for modeling pyrolysis behavior, as variables such as ash, volatile matter, and elemental ratios can interact in complex, multidimensional ways. NMS was used to investigate wide parameter ranges for both DNN and LightGBM prior to fine-tuning^35^.

#### Puma Optimizer (PO)

The Puma Optimizer (PO) is a cooperative-search metaheuristic inspired by coordinated pursuit dynamics. It achieves a compromise between a broad search of the space with a quick and efficient convergence, thus allowing a fast refinement of the promising hyperparameter regions that have been located^36^. PO was the main instrument of the third stage of tuning used to fine-adjust those hyperparameters that had a high impact, e.g., learning rate, tree depth, and neuron-layer configurations. Its stability and convergence resilience made it a perfect partner to NMS in the coupled two-step optimization scheme employed in this work^37^.

#### Integration with machine learning models

Selection of NMS and PO were both linked to cross-validated performance metrics in order to ensure that changes of hyperparameters were valid beyond the training subset. Several parameters were optimized for the DNN, including learning rate, dropout rate, number of neurons per layer, batch size, and activation-related settings. The optimizers for LightGBM tuned the key structural parameters, which are num_leaves, max_depth, feature_fraction, bagging_fraction, and learning_rate. Each optimizer independently produced a different tuned version of both models, so four calibrated predictive frameworks were compared in section “Results and discussion”. The use of these two optimizers means that the final comparisons between DNN and LightGBM reflect the models’ capabilities rather than the choices of suboptimal hyperparameters, thus allowing fair benchmarking and scientifically interpretable conclusions.

### Performance evaluators

The model’s efficiency was evaluated in this study using several performance measures, including mean square error (MSE), root mean square error (RMSE), BIAS, coefficient of determination (R^2^), and the ratio of RMSE to standard deviation (RSR). Below are the formulas for these performance standards:

1 $${R^2}={\left( {\frac{{\mathop \sum \nolimits_{{i=1}}^{n} \left( {{b_i} - \bar {b}} \right)\left( {{m_i} - \bar {m}} \right)}}{{\sqrt {\left[ {\mathop \sum \nolimits_{{i=1}}^{n} {{\left( {{b_i} - \bar {b}} \right)}^2}} \right]\left[ {\mathop \sum \nolimits_{{i=1}}^{n} {{\left( {{m_i} - \bar {m}} \right)}^2}} \right]} }}} \right)^2}$$

2 $$RMSE=\sqrt {\frac{1}{n}\mathop \sum \limits_{{i=1}}^{n} {{\left( {{m_i} - {b_i}} \right)}^2}}$$

3 $$MSE=\frac{1}{n}\mathop \sum \limits_{{j=1}}^{n} {\left( {{m_i} - {b_i}} \right)^2}$$

4 $$BIAS=\bar{b} - \bar{m}$$

5 $$RSR=\frac{{RMSE}}{{St.D}}$$

where the sample size is denoted by $n$. The predicted value is represented by ${b}_{i}$. $\bar{m}$ and $\bar{b}$, respectively, stand for the measured and mean predicted values. The measured value is denoted by ${m}_{i}$.

## Results and discussion

The evidence in this section serves the purpose of evaluating the main hypothesis of the research, which states that a chemically educated feature set, along with sophisticated nonlinear learning architectures, can better explain the thermochemical mechanisms of bio-oil formation than conventional regression or untuned ensemble models. The dataset, which combines measurements from different studies with varying biomass compositions, reactor conditions, and analytical protocols, makes the predictive task not just a simple one of curve fitting, but rather a task that requires a model capable of generalizing across different experimental sources. Therefore, the investigation is centered around the question of whether the physico-chemical features that were engineered, the VIF-refined input matrix, and the stratified training scheme together allowed deep learning and optimized boosting models to discover significant patterns that correspond to devolatilization behavior, ash-catalyzed secondary reactions, and elemental reactivity trends. The subsections below evaluate these models not only in terms of their numerical performance but also in terms of their capacity to offer chemically interpretable and cross-study-robust predictions of bio-oil yield.

### Outlier analysis and distribution inspection

**Fig. 3:**
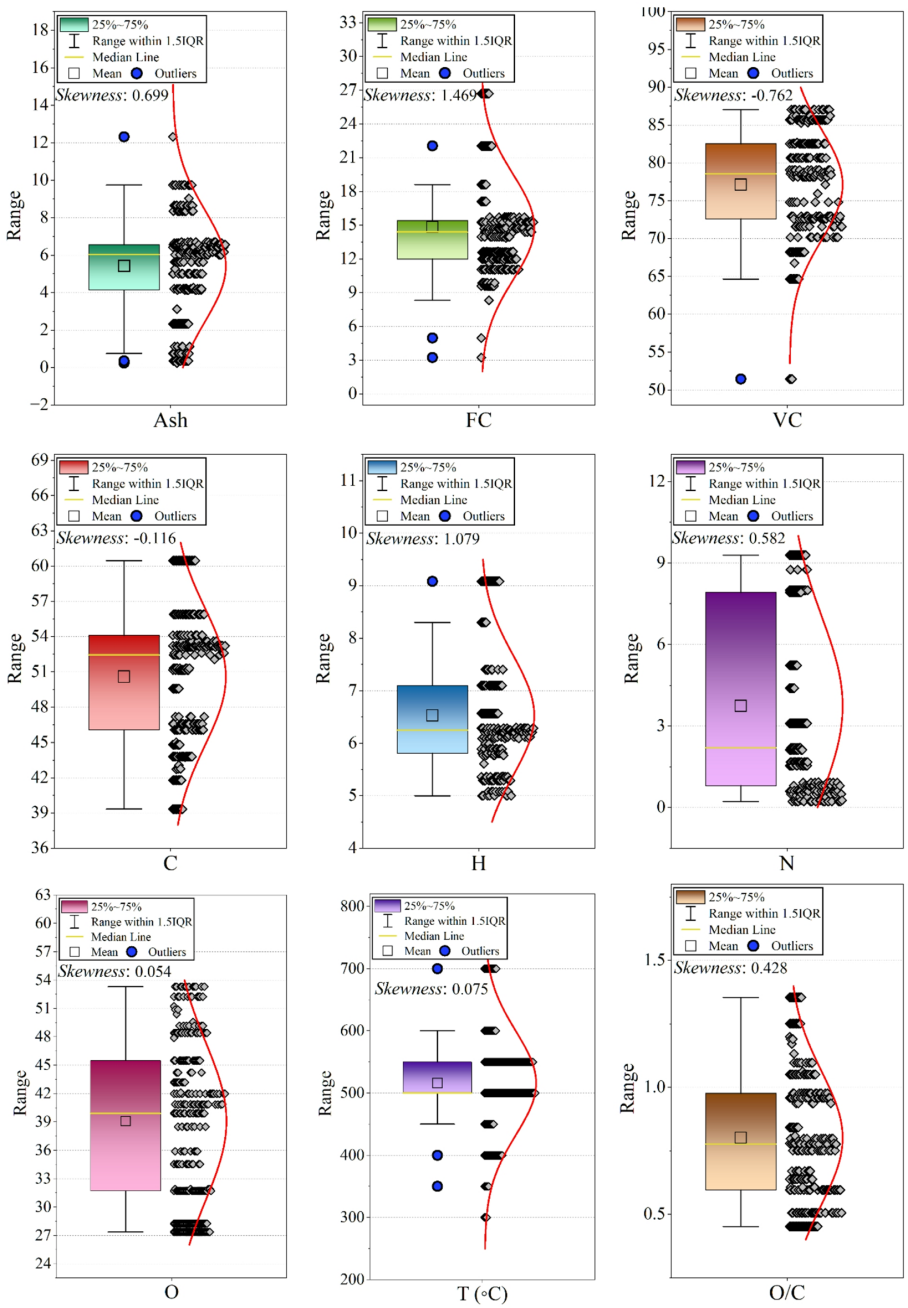

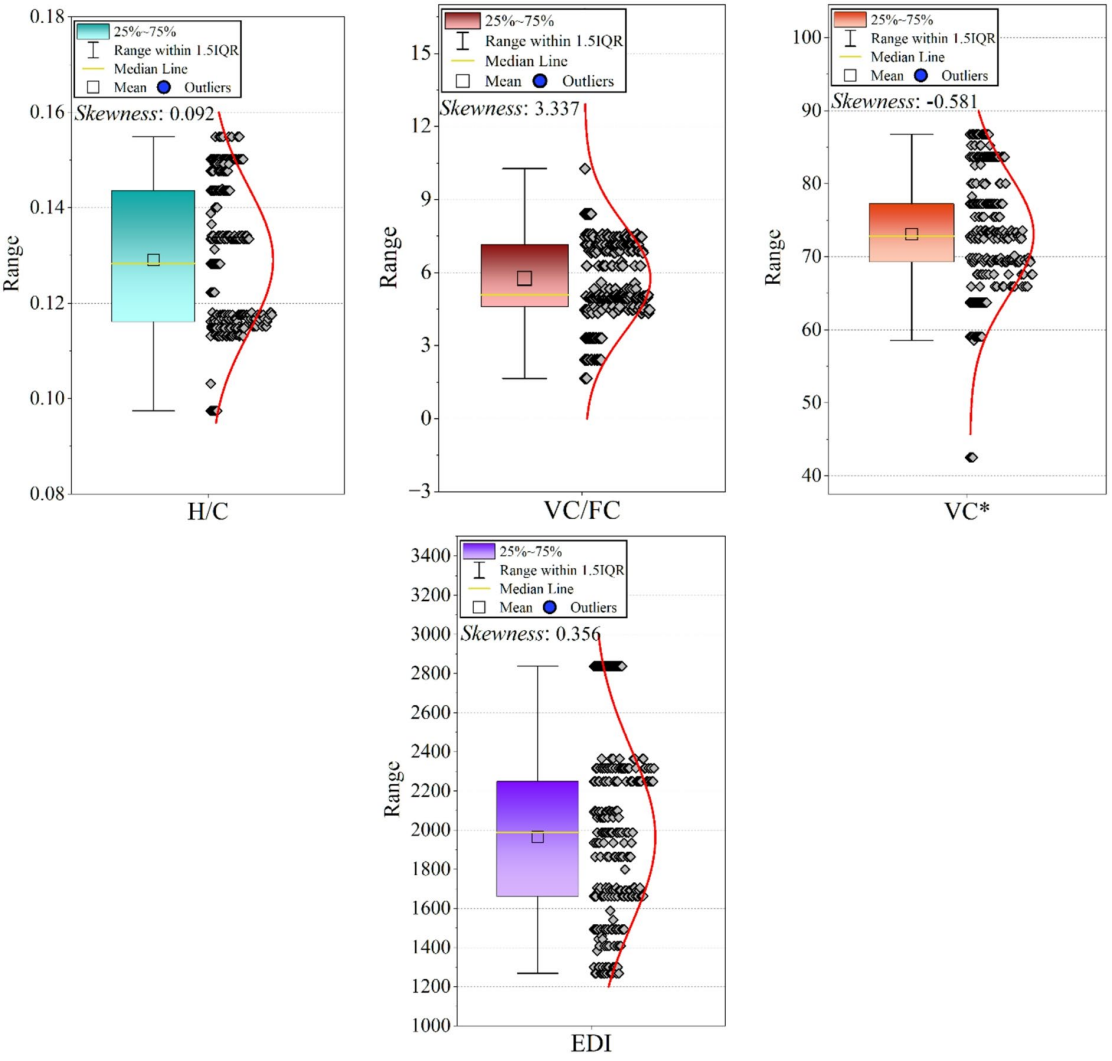
A grid of violin/box plots showing the distribution of each variable (original and engineered features). This visualizes skewness, spread, and potential outliers.

Distribution plots (Fig. 3) were developed for all variables to identify extreme values arising from measurement differences across referenced studies. No synthetic removal was performed, but extreme values were flagged and included as potential drivers of nonlinear behavior.

### Final input feature matrix and multicollinearity assessment

**Fig. 4:**
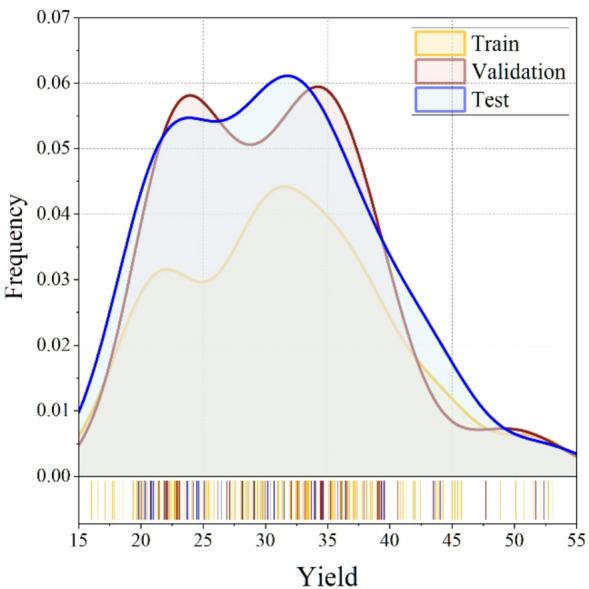
A three-panel distribution comparison plot (train/validation/test) showing the kernel density estimates of bio-oil yield, demonstrating uniform coverage across subsets.

In order to ensure both statistical stability and physically meaningful predictions, the present study carried out a thorough multicollinearity assessment for the engineered features using the Variance Inflation Factor (VIF). Given that engineered features are derived from the original variables, it was deemed more appropriate to exclude original features from the VIF analysis. This approach allows us to focus on the direct relationships between the engineered features and the target variable (bio-oil yield), without introducing unnecessary redundancy. Table 2 presents the VIF values for the engineered features and the feature selection process followed from this analysis. The engineered variables were selected based on their ability to capture thermochemical and process-related information without exceeding acceptable collinearity thresholds. Features with high VIF and limited predictive value were excluded. After this assessment, the final feature matrix retained 9 features, including a mixture of proximate analysis variables, elemental composition, pyrolysis conditions, and key engineered ratios. These variables provided a balanced representation of the biomass feedstock’s chemistry and the pyrolysis process’s behavior. The samples were partitioned into three subsets: 70% for training, 15% for validation, and 15% for testing (Fig. 4). To ensure that the distribution of each feature was also consistent in the three partitions, distribution-similarity evaluations were performed using statistical overlap scores. These diagnostics confirmed that no subset displayed an over-representation of particular biomass types or temperature ranges, thus facilitating generalizable and interpretable model performance.

**Table 2:** VIF for original and engineered features.

Feature	Type	VIF	Interpretation	Final selection
Ash	Original	2.4	Low collinearity; relatively independent mineral fraction	Selected
Fixed Carbon (FC)	Original	5.8	Moderate correlation with volatile matter; expected in proximate analysis	Selected
Volatile Content (VC)	Original	6.9	Moderately high collinearity; inversely related to FC	Excluded (high VIF)
Carbon (C)	Original	3.1	Limited correlation with proximate analysis variables	Selected
Hydrogen (H)	Original	2.7	Independent across feedstocks; low redundancy	Selected
Nitrogen (N)	Original	1.5	Minimal collinearity; low variability across samples	Excluded (low VIF but later excluded due to negligible predictive contribution)
Oxygen (O)	Original	4.4	Some correlation with VC and C, but acceptable	Selected
Pyrolysis Temperature (T)	Original	1.2	Independent process variable	Selected
H/C Ratio	Engineered	7.9	High collinearity with C and H; expected for derivative metrics	Selected
O/C Ratio	Engineered	8.6	High collinearity with O and C; expected for derivative metrics	Excluded (high VIF + high redundancy with O and C)
VC/FC Ratio	Engineered	9.3	Strongly collinear with VC and FC; captures the same underlying structure	Excluded (high VIF + behavior captured by VC and FC individually)
Ash-Corrected VC (VC*)	Engineered	6.8	Correlated with VC and Ash, but remains below the critical threshold	Selected
Energy Density Index (EDI)	Engineered	4.9	Moderate correlation with elemental composition	Selected

### K-fold cross-validation

Table 3 reports the performance of the DNN and LGB models across ten folds using RMSE and R^2^ as evaluation metrics. Overall, the DNN consistently outperforms LGB across all folds, achieving substantially lower RMSE and higher R^2^, indicating superior predictive accuracy and better goodness-of-fit. For the DNN, RMSE values range from 1.7891 (K10) to 2.5487 (K3), while R^2^ values range from 0.9053 (K3) to 0.9539 (K10). This relatively narrow spread demonstrates the DNN’s robustness and stability across different data partitions. The best DNN performance is observed in K10, where the lowest RMSE and highest R^2^ are obtained, whereas K3 represents the most challenging split, exhibiting the highest RMSE and lowest R^2^. Despite this variability, the DNN maintains strong performance in all folds, with R^2^ consistently above 0.90. In contrast, LGB shows noticeably weaker and more variable results. Its RMSE ranges from 3.5165 (K10) to 3.9831 (K3), and R^2^ spans from 0.8702 (K3) to 0.9087 (K10). Similar to DNN, LGB also performs worst in K3, suggesting that this fold likely contains more complex or less representative samples. However, even in its best case (K10), LGB does not achieve the accuracy of DNN. Comparing the folds, K10 consistently yields the best results for both models, while K3 yields the worst, indicating that data composition in these folds has a tangible impact on model accuracy. Across all folds, DNN improves RMSE by roughly 1.3–2.0 units relative to LGB and increases R^2^ by approximately 0.03–0.07, highlighting a clear and consistent performance margin.

**Table 3:** Result of the K-fold cross-validation.

Fold	Model	Metrics	
		RMSE	R^2^
K1	DNN	2.0946	0.9362
	LGB	3.6299	0.9003
K2	DNN	2.3447	0.9198
	LGB	3.5744	0.9038
K3	DNN	2.5487	0.9053
	LGB	3.9831	0.8702
K4	DNN	2.1253	0.9340
	LGB	3.5218	0.9072
K5	DNN	2.0226	0.9408
	LGB	3.6196	0.9015
K6	DNN	2.1098	0.9355
	LGB	3.8239	0.8842
K7	DNN	2.0468	0.9389
	LGB	3.5935	0.9022
K8	DNN	2.2886	0.9234
	LGB	3.9596	0.8719
K9	DNN	1.9924	0.9422
	LGB	3.6154	0.9065
K10	DNN	1.7891	0.9539
	LGB	3.5165	0.9087

### Hyperparameter analyses

Table 4 presents the final hyperparameter configurations for the DNN and LightGBM models under the baseline setting, NMS, and PO. For both model families, an identical optimization budget of 200 iterations was enforced to guarantee a fair comparison across optimizers. Each optimizer used the same population size (20 candidate solutions), identical initialization ranges, and the same objective function (minimization of validation RMSE), ensuring that performance differences arise from optimizer dynamics rather than unequal search effort.

For the deep neural network, the tuned hyperparameters included the number of neurons per hidden layer, the learning rate, the batch size, and the number of epochs. The search ranges were defined as follows: neurons per layer ∈ [32, 256], learning rate ∈ [10^−4^, 5 × 10^−2^], batch size ∈ [16, 64], and epochs ∈ [20, 150]. PO converged to a deeper, wider architecture (64–128–128 neurons), combined with a higher number of training epochs (140) and a moderately increased learning rate (0.0153), compared with both the baseline DNN and the NMS-optimized variant. In contrast, NMS favored a shallower structure with fewer epochs and a higher learning rate. The DNPO configuration indicates that PO systematically explored higher-capacity networks and longer training schedules, consistent with its superior predictive accuracy reported in Table 3.

For LightGBM, the tuned parameters were num_leaves, max_depth, learning_rate, n_estimators, and max_bin, with search ranges defined as num_leaves ∈ [20, 1000], max_depth ∈ [− 1, 1000], learning_rate ∈ [0.01, 1.0], n_estimators ∈ [50, 200], and max_bin ∈ [200, 10000]. The PO-optimized LightGBM (LGPO) selected structurally large but balanced configurations (num_leaves = 305, max_depth = 983, max_bin = 8300) with a moderate ensemble size (97 trees). By contrast, NMS pushed several parameters toward extreme values (e.g., num_leaves = 999 and learning_rate = 0.999), indicating stronger sensitivity to initialization and a tendency toward premature exploitation.

Although both NMS and PO were allocated the same number of iterations and population size, PO demonstrated more consistent convergence toward stable, physically plausible hyperparameter combinations, whereas NMS exhibited greater variability across runs, particularly with LightGBM. This behavior reflects the stronger exploration–exploitation balance embedded in PO, leading to improved robustness with respect to initialization. Consequently, the superior performance of DNPO and LGPO cannot be attributed to increased computational effort but rather to the intrinsic optimization dynamics of the Puma Optimizer.

All models were implemented in Python using TensorFlow/Keras (v2.13) for deep learning, LightGBM (v4.1) for gradient boosting, and NumPy (v1.24) and scikit-learn (v1.3) for preprocessing, data splitting, and evaluation metrics. The metaheuristic optimizers (NMS and PO) were implemented using custom Python routines integrated with these libraries. Hyperparameter tuning was conducted using the same training–validation split for all optimizers, and the final reported performances correspond to retraining with the selected optimal parameters followed by evaluation on the held-out test set. This unified experimental protocol ensures transparency, reproducibility, and an unbiased comparison between optimization strategies.

**Table 4:** The hyperparameters of the hybrid models and their corresponding values.

Hyperparameter	Hybrid models					
	DNN	DNNM	DNPO	LGB	LGNM	LGPO
Number of neurons per layer	128,64,32	96,128,48	64,128,128	–	–	–
Learning rate	0.001	0.0194	0.0153	–	–	–
Batch size	32	48	32	–	–	–
Epochs	20	80	140	–	–	–
Num leaves	–	–	–	31	999	305
Max depth	–	–	–	−1	199	983
Learning rate	–	–	–	0.1	0.999	0.4103
N estimators	–	–	–	100	199	97
Max bin	–	–	–	200,000	1999	8300

### Evaluation of deep learning and gradient boosting frameworks

The performance metrics in Table 5 reveal the same clear and consistent trends for all phases: training, validation, and testing. These results each illustrate the major differences between the models in their capacity to represent the nonlinear thermochemical dynamics that dictate bio-oil yield. The performance of the Deep and Boosting-based hybrid models was evaluated across training, validation, and testing phases using multiple statistical indices, including RMSE, R^2^, MSE, RSR, and BIAS. Among the boosting-based models, the LightGBM with parameter optimization (LGPO) consistently outperformed the other variants, achieving lower RMSE values and higher R^2^ across all phases. Specifically, LGPO attained a test RMSE of 2.038 and R^2^ of 0.938, indicating strong predictive capability. In contrast, the standard LightGBM (LGB) exhibited the poorest performance, with higher errors and inconsistent bias, particularly in the validation phase (RMSE = 3.507, R^2^ = 0.873, BIAS = −0.336), suggesting potential overfitting and reduced reliability without parameter optimization. The deep learning models demonstrated superior performance compared to the boosting-based models. The deep neural network with parameter optimization (DNPO) achieved the lowest RMSE and highest R^2^ across all phases, with a test RMSE of 1.138 and R^2^ of 0.980, indicating excellent predictive accuracy. Furthermore, DNPO exhibited minimal bias (BIAS = −0.003) and the lowest RSR value (0.143), reflecting both high accuracy and stability. The other deep learning variants, DNNM and DNN, also provided strong predictions, with test RMSE values of 1.640 and 1.574, respectively, and R^2^ above 0.95, further confirming the robustness of deep models in capturing complex patterns within the dataset. Comparing the models across phases reveals that deep learning models, particularly DNPO, maintain consistent performance from training to testing, demonstrating good generalization capabilities and minimal overfitting. In contrast, some boosting-based models, especially LGB, showed larger discrepancies between training and validation results, highlighting the importance of parameter tuning for reliable predictions. Overall, the results indicate that the optimized deep neural network (DNPO) provides the most accurate and unbiased predictions, making it the preferred model for the target application.

**Table 5:** Evaluation result of the deep and boosting-based hybrid models.

Phase	Model	Index values				
		RMSE	R^2^	MSE	RSR	BIAS
Train	LGPO	2.010	0.944	4.039	0.238	0.002
	LGNM	2.371	0.928	5.624	0.280	0.318
	LGB	3.688	0.912	13.600	0.436	−0.025
	DNPO	1.036	0.985	1.074	0.123	−0.015
	DNNM	1.505	0.969	2.264	0.178	−0.022
	DNN	1.769	0.957	3.129	0.209	−0.029
Validation	LGPO	2.230	0.913	4.972	0.297	0.197
	LGNM	2.546	0.887	6.483	0.339	0.186
	LGB	3.507	0.873	12.296	0.467	−0.336
	DNPO	1.593	0.956	2.539	0.212	−0.082
	DNNM	1.838	0.941	3.379	0.245	−0.175
	DNN	2.246	0.912	5.045	0.299	−0.275
Test	LGPO	2.038	0.938	4.155	0.255	0.439
	LGNM	2.303	0.922	5.305	0.289	−0.097
	LGB	3.433	0.921	11.788	0.430	0.462
	DNPO	1.138	0.980	1.295	0.143	−0.003
	DNNM	1.640	0.958	2.689	0.205	0.125
	DNN	1.574	0.962	2.476	0.197	0.010

The convergence metrics in Table 6 illustrate the changes in the models that were optimized in terms of their reliability and stability. RMSE statistics, namely, minimum, mean, and maximum, depict the consistency with which each optimization approach reaches a low-error region in the search space. In all cases, the DNPO model displays the most advantageous and stable convergence behavior. DNPO not only achieves the lowest minimum RMSE (1.1523) but also maintains the lowest mean RMSE (1.7566), indicating that the PO consistently guides the DNN toward a high-quality solution. Compared with the DNPO model, LightGBM can achieve RMSEs that are even higher (5.2627). The DNNM model has minimum and mean RMSE values that are only slightly higher than those of DNPO, indicating that the NMS optimizer is less efficient at identifying hyperparameter combinations for deep learning. On the other hand, the maximum RMSE is the same as that of DNPO, signaling that once NMS goes into a bad region of the search space, both optimizers face similar upper-bound performance limitations, probably due to the intrinsic nature of the DNN architecture. The LightGBM models have wider spreads of errors. LGPO is a condition that PO enhances. The mean RMSE is 3.0573, whereas the maximum is 6.4647, which points to the fluctuation in convergence. This situation is well understood: setting the num_leaves and tree depth parameters incorrectly can have a significant impact on error; thus, LightGBM is sensitive to hyperparameters. Small configuration changes can lead to big errors. LGNM takes this trend even further and has the highest minimum, mean, and maximum RMSE values, which confirms that NMS has a hard time finding its way through the most irregular optimization surface that comes from gradient-boosted trees.

**Table 6:** Descriptive statistical summary of convergence metrics (minimum, mean, and maximum).

Descriptor	Models			
	LGPO	LGNM	DNPO	DNNM
Min RMSE	2.0486	2.3889	1.1523	1.5795
Mean RMSE	3.0573	3.4317	1.7566	2.1118
Max RMSE	6.4647	6.9782	5.2627	5.2627
Total iterations	200			

**Fig. 5:**
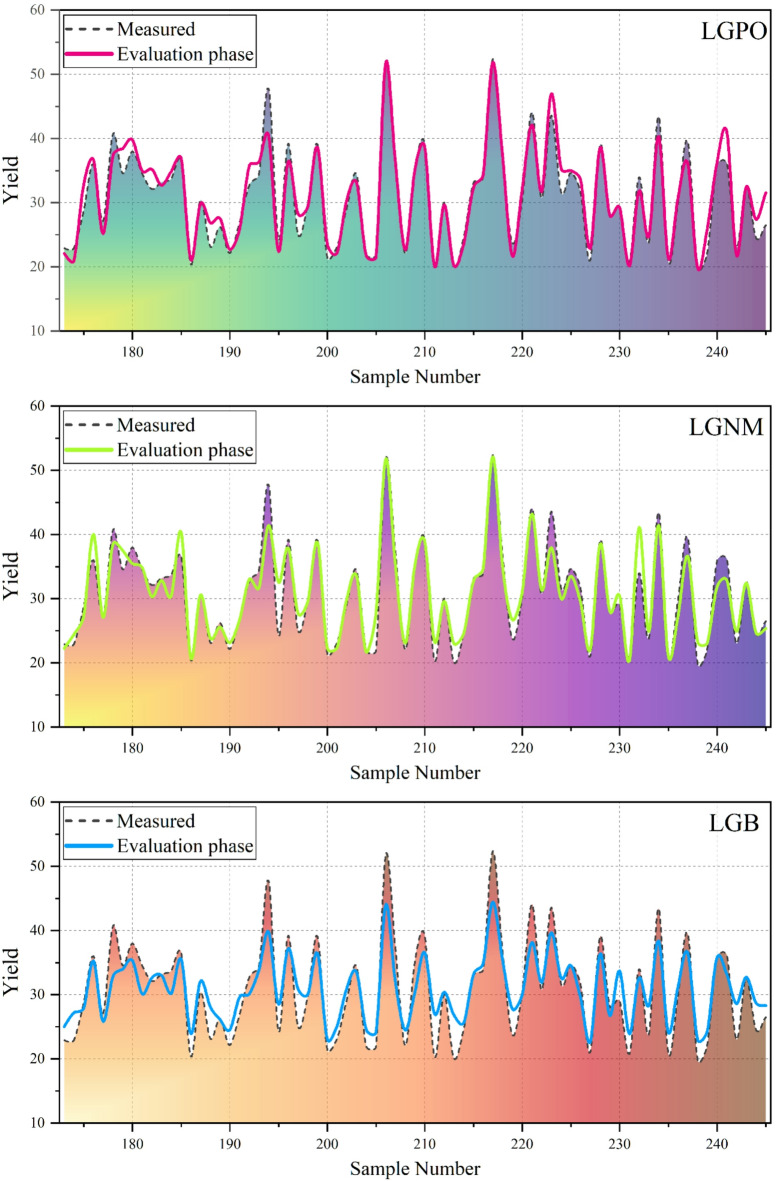

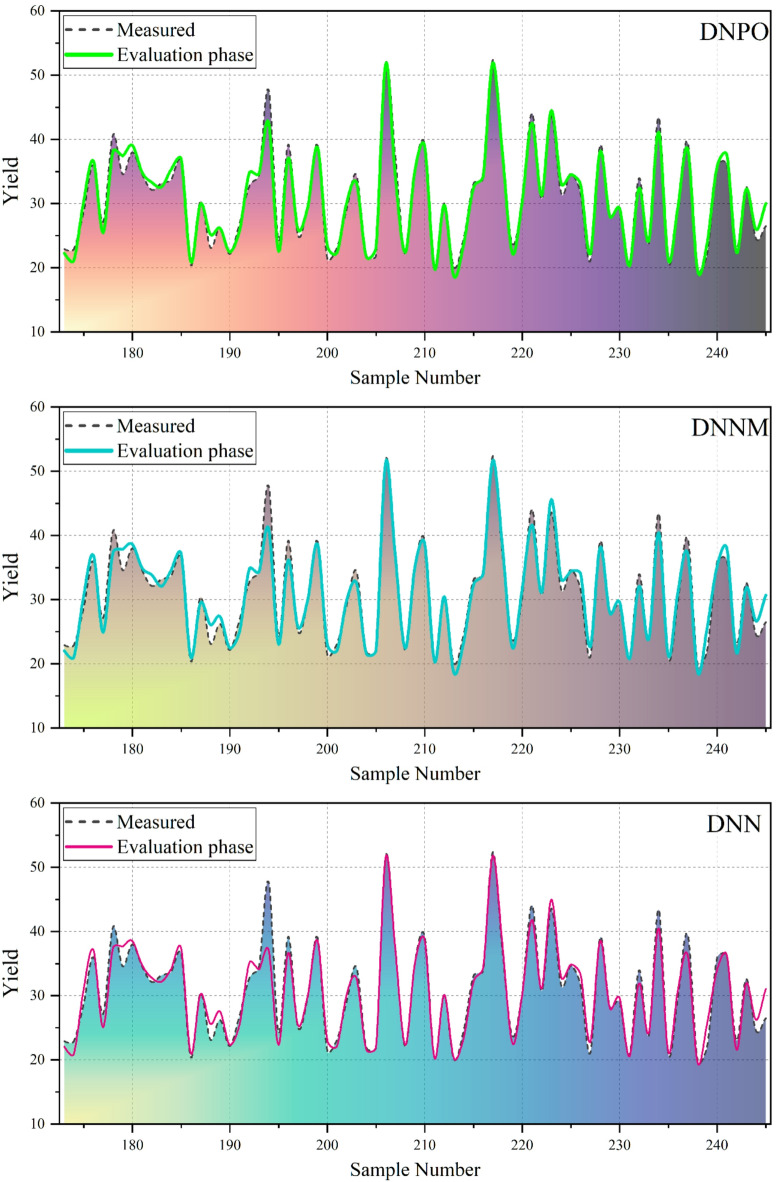
The comparison of predicted and measured values of the models based on the line-symbol plot.

Figure 5 shows a comparison of bio-oil yield obtained from experiments and those predicted by the developed models over the evaluation samples. Both models were able to capture the overall oscillatory pattern of the experimental data that reflects a successful reconstruction of the nonlinear volatility of biomass pyrolysis. However, there are distinct differences in how close each model follows the peak and valley transitions. The DNPO model demonstrates very good correspondence with the measured values, particularly in regions where the experimental yield changes abruptly. Its prediction curve is very close to both local maxima and minima, with little time lag, DNPO can be strongly considered to respond to variations driven by compositional changes and temperature. The small departures at a few peak points of the high yields suggest minor smoothing effects, but overall, the DNPO keeps a tight fit over the entire sample range. The DNNM model performs well, but it is still slightly affected by deviations in the peak regions. There are times when the predicted values under-estimate that point when the yield is high and over-estimate that point where the yields are low and close to the high-yield points, which indicates that the changes in feedstock characteristics cannot be detected abruptly. However, the general pattern remains good, and the baseline yields are accurate throughout the series.

### Statistical significance analysis using the Wilcoxon test

Differences in the predictive performance of the models that were developed were parameterized using the Wilcoxon signed-rank test presented in Table 7. Since all models were tested on the same data partitions, this nonparametric paired test is used to identify any systematic differences in their prediction distributions. The results of the experiment clearly indicate the pattern: models that use global optimization or deeper nonlinear architectures usually differ significantly from baseline or lightly tuned models, thus confirming that the improvements are not just random fluctuations reported earlier. LightGBM variants LGPO versus LGNM (*p* = 0.142) show no significant difference, which means that, although PO slightly improves performance over NMS, the predictive distributions overlap substantially. On the other hand, LGPO versus DNNM (*p* = 0.0219) and LGPO versus LGBM (*p* = 0.0059) differ significantly. The evidence confirms that (i) optimized LightGBM is different from its untuned counterpart and (ii) the DNN architecture introduces nonlinear learning dynamics that are not captured by gradient boosting. Besides, when comparing LGNM with DNPO, significant differences are found (*p* = 0.0422) and LGBM (*p* = 0.0313), indicating that the optimization and model complexity have an impact on predictive structure in a significant way.

On the other hand, a few of the LASSO comparisons, e.g., with DNPO (*p* = 0.5794), DNNM (*p* = 0.5647), and LGBM (*p* = 0.4041), are not statistically significant. LASSO’s limited capacity to represent the data results in higher but more uniform errors, which sometimes overlap with the more flexible models, thus yielding non-significant pairwise differences despite weaker performance. Last but not least, DNPO versus DNNM (*p* = 0.7386) is also a non-significant difference, consistent with the fact that both models have the same neural architecture and differ only in optimized hyperparameters.

**Table 7:** Result of the Wilcoxon test.

Difference between models	Parameter	
	*p*_value	Statistic
Def. between LGPO and LGNM	0.1420	13,437
Dif. between LGPO and LASSO	0.8370	14,839
Dif. between LGPO and DNPO	0.1440	13,445
Dif. between LGPO and DNNM	0.0219	12,523
Dif. between LGPO and LGBM	0.0059	12,008
Dif. between LGNM and LASSO	0.5775	14,449
Dif. between LGNM and DNPO	0.0422	12,812
Dif. between LGNM and DNNM	0.0807	13,128
Dif. between LGNM and LGBM	0.0313	12,677
Dif. between LASSO and DNPO	0.5794	14,452
Dif. between LASSO and DNNM	0.5647	14,428
Dif. between LASSO and LGBM	0.4041	14,141
Dif. between DNPO and DNNM	0.7386	14,697

### Comparison with the published study

Table 8 compares the presented and published studies. The Hybrid DNPO model outperforms previous approaches, with an RMSE of 1.138 and an R^2^ of 0.980, indicating superior predictive accuracy and better model fit than those reported in other studies. While some studies, such as Onokwai et al.^19^, achieved low RMSE values, their models are often specific to particular feedstocks, such as Cocos nucifera, limiting their applicability to other types of organic solid waste. The current framework’s broader scope and enhanced predictive capabilities make it more versatile for a range of feedstocks. However, despite its advantages, the Hybrid DNPO model still faces challenges in generalizing across all types of feedstocks, as evidenced by the limitations of the models discussed. The reliance on specific biochemical features and operating conditions in some of these studies may limit their ability to predict bio-oil yields for more diverse or novel feedstocks accurately.

**Table 8:** Comparison between the presented and published studies.

Study	References	Dataset size	Feature set	Model	RMSE	*R* ^2^
Present study		245	Proximate + Ultimate + Engineered	DNPO	1.138	0.980
Djandja et al.	^18^	150	Biochemical + Operating Factors	XGB	–	0.944
Onokwai et al.	^19^	120	Temperature, Heating Rate	PSO-ANFIS	0.449	0.994
Mathur et al.	^38^	200	Proximate + Ultimate	GB	2.39	0.97
Ortiz-Alvarez et al.	^39^	180	Proximate + Ultimate	ANN	1.310	0.9739

### Sensitivity analysis of key physicochemical features

**Fig. 6:**
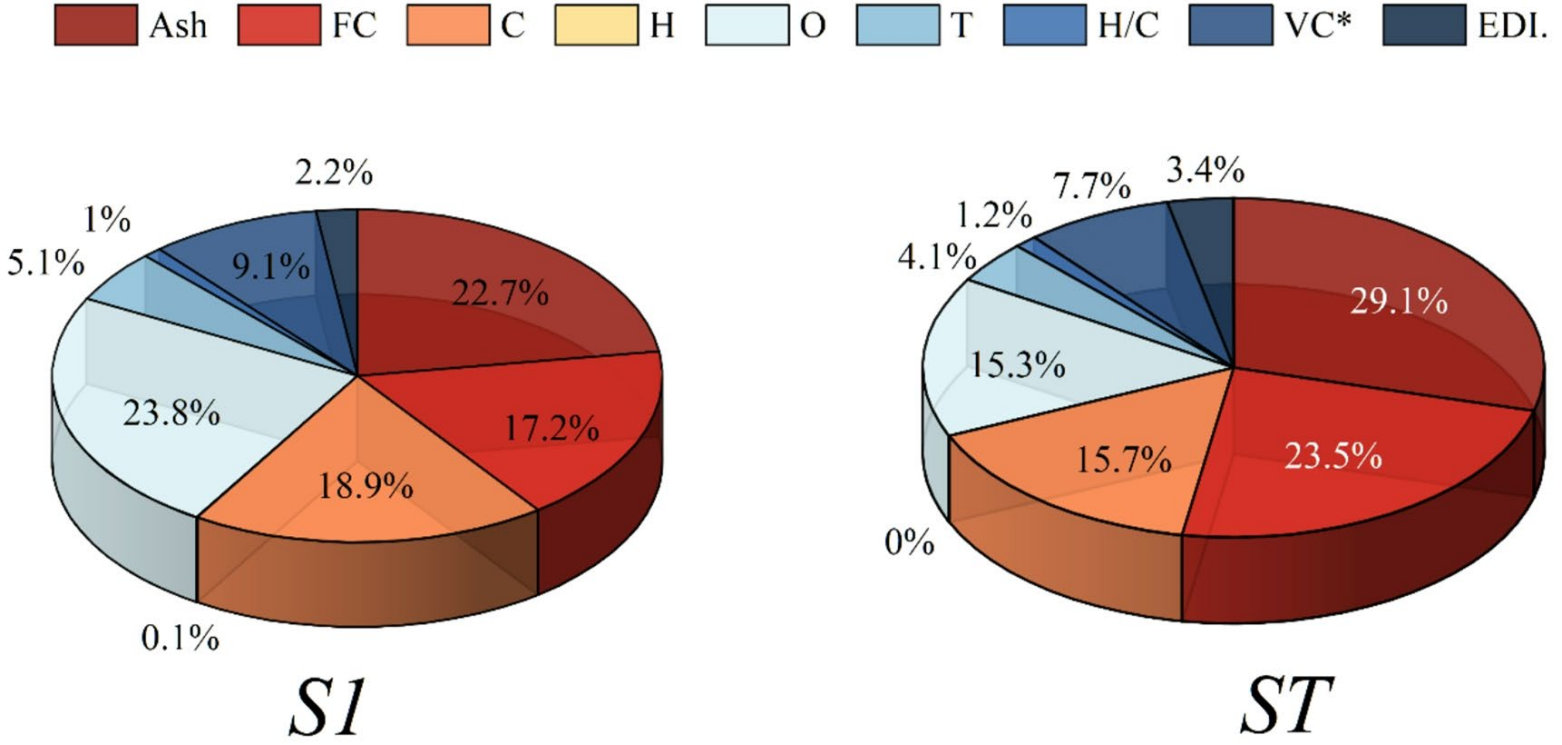
The FAST sensitivity analysis of the best-performing model.

The FAST sensitivity analysis from Fig. 6 unveils the manner in which each physicochemical property impacts the prediction of bio-oil yield based on the model that has been optimized. The S1 indices (left chart) measure the direct, independent effect of each feature, while the ST indices (right chart) demonstrate the combined effect, including all interaction influences. Looking at the S1 results, one can see that carbon content (C) is the most influential direct predictor (23.8%), which aligns with its pivotal role in determining devolatilization behavior and the intrinsic energy density of biomass. Fixed carbon (FC) and ash follow with 22.7% and 18.9%, respectively. Their strong contributions are well aligned with the aforementioned thermochemical trends: fixed carbon is responsible for secondary charring pathways, whereas ash, enriched in alkali and alkaline-earth metals, accelerates cracking and alters volatile release patterns. Temperature (T) is not left behind in exerting a significant influence (17.2%), due to its nonlinear control over primary pyrolysis kinetics. The elements hydrogen and oxygen, along with features derived from them such as H/C and VC*, are making smaller but still significant contributions, meaning that molecular composition gradients still contain some information, although less independent than the elemental backbone variables.

However, the ST indices indicate a substantial change. Carbon (29.1%) and fixed carbon (23.5%) now together explain an even larger part of total variance, thus defining the strong interaction effects with temperature, oxygen content, and ash composition. The role of temperature is further highlighted as its contribution increases from 17.2 to 15.7%, underscoring its synergistic effect with compositional features during volatilization and secondary reactions once again. One of the largest increases that ash shows is (from 18.9 to 23.5%), which is a confirmation that the mineral-induced catalytic effects interact greatly with other feed attributes. At the same time, interaction-sensitive features such as VC* and H/C that show slight increases in ST are consistent with their role in mediating compositional–thermal coupling rather than exerting dominant direct effects.

## Conclusion

The investigation reported here is a clear example of how integrating chemically aware feature engineering with state-of-the-art machine learning methods opens a trustworthy avenue for predicting bio-oil yield from diverse biomass and pyrolysis conditions. By moving beyond traditional feature matrices and using elemental composition, thermochemical indicators, and engineered physicochemical descriptors, the framework effectively captures the complex nonlinearities that typical models fail to address. The deep neural network with Puma Optimizer (DNPO) was the model that consistently achieved the closest approximation to the true predictive output across the training, validation, and test data sets among all the methods tested. Its superior performance reflects both its ability to learn multiscale interactions and the optimization strategy’s efficiency in dealing with a difficult hyperparameter space. LightGBM models, on the other hand, although close, were less stable and more dependent on optimization changes. The global sensitivity analysis, using the FAST method, identified carbon content, fixed carbon, ash content, and pyrolysis temperature as the key factors influencing bio-oil yield prediction. This aligns with the expected thermochemical behavior of biomass and enhances the model’s interpretability. Furthermore, the study sets a new benchmark by combining feature engineering, optimization-aware modeling, and statistical validation through convergence metrics, Wilcoxon tests, and global sensitivity indices. While the findings are promising, they are limited by the size and heterogeneity of the dataset derived from the literature. Future work should focus on using larger experimental datasets, quantifying uncertainty, and conducting model-guided pyrolysis experiments to improve our understanding of the cause-and-effect relationships in bio-oil production. Extending this framework to mechanistic hybrid models or physics-informed neural networks could further improve interpretability. In conclusion, the data-centric, optimization-enhanced learning models demonstrated here have the potential to significantly improve process design, yield estimation, and decision-making in the sustainable production of biofuels. In future studies, a comparison with first-principles models based on the kinetic models of pyrolysis will be carried out. First-principles models rely on known physical and chemical mechanisms to predict bio-oil yield, but they are typically less flexible than data-driven approaches. These models have been used for predicting pyrolysis behavior under specific conditions, providing insight into reaction mechanisms and energy balances, but they lack the ability to accommodate more complex feedstock types or capture broader variability seen in experimental data. While first-principles models provide mechanistic insights, data-driven models like Hybrid DNPO offer superior predictive accuracy. The comparison between these two approaches will highlight the trade-offs between flexibility and interpretability, and help determine the best approach depending on the context. In addition, the model will be tested on unseen experimental datasets that lie outside the original range of the training data. A comparison with first-principles models will also be conducted to demonstrate the advantages and limitations of both approaches. Finally, the literature review will be expanded to cover a broader set of studies, strengthening the context and justification for the proposed methodology.

## Supplementary Information

Below is the link to the electronic supplementary material.

Supplementary Material 1

## Data Availability

The data supporting the findings of this study are available from the corresponding author upon reasonable request.
